# Comparison of room times between pulsed-field ablation and very high-power short-duration ablation

**DOI:** 10.1016/j.hroo.2025.07.008

**Published:** 2025-07-18

**Authors:** Gábor Orbán, Márton Boga, Zoltán Salló, István Osztheimer, Klaudia Vivien Nagy, Péter Perge, Edit Tanai, Bence Czumbel, Bertalan Bakán, Ferenc Komlósi, Patrik Tóth, Arnold Béla Ferencz, Béla Merkely, László Gellér, Nándor Szegedi

**Affiliations:** Heart and Vascular Center, Semmelweis University, Budapest, Hungary

**Keywords:** Atrial fibrillation, Room time, Pulsed-field ablation, Very high-power short-duration ablation, Time management, Procedural efficiency

## Abstract

**Background:**

Pulsed-field ablation (PFA) has shown promise in improving atrial fibrillation (AF) ablation efficiency by reducing skin-to-skin procedure times while maintaining safety compared with advanced radiofrequency methods such as very high-power short-duration (vHPSD) ablation. Although PFA requires deeper sedation, its effect on total time spent in the operating room is unknown.

**Objective:**

We aimed to compare the room time (the time between operating room entry and exit) and procedural subsections’ times of PFA and vHPSD procedures.

**Methods:**

We enrolled consecutive patients who underwent PFA or vHPSD ablation at our center. Room and procedural subsections’ times were analyzed. Recurrence rates at 6 months were also compared.

**Results:**

We included 131 patients (66 [55–71] years, 86 [65.6%] had paroxysmal AF). Eighty-seven patients (66%) underwent PFA, and 44 (34%) underwent vHPSD ablation. PFA outperformed vHPSD in terms of room time (71 [64–80] vs 88 [75–99.8] minutes, *P* < .001) and in most procedural subsections. One major nonfatal complication occurred with PFA, whereas no major complication occurred with vHPSD. There was no significant difference in 6-month recurrence rates between the 2 groups (PFA 15%, vHPSD 18%, *P* = .646).

**Conclusion:**

In AF ablation, PFA results in significantly shorter room time than vHPSD, while maintaining similar 6-month recurrence rates. PFA may enhance time efficiency in the electrophysiology laboratory, even compared with the fastest radiofrequency technology.

## Graphical abstract

**Figure:**
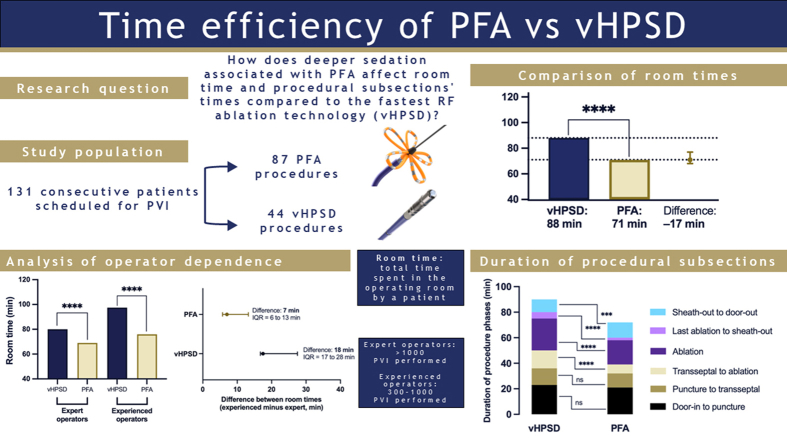

**Key Findings**

▪ Despite requiring deeper sedation, our study shows that pulse-field ablation (PFA) significantly reduces total room time (defined as the total time spent in the operating room by a patient, including anesthesia induction and emergence period) compared with the fastest radiofrequency technology (very high-power short-duration [vHPSD]).
▪ PFA’s total room time is 17 minutes shorter than vHPSD’s room time, without compromising 6-month recurrence rates.
▪ PFA is less operator dependent in total room time than vHPSD; therefore, PFA minimizes variability linked to operator experience.

## Introduction

Atrial fibrillation (AF) is the most common sustained cardiac arrhythmia with increasing prevalence, which places a significant burden on health care systems worldwide.^1^ Pulmonary vein isolation (PVI) has emerged as a pivotal and effective therapeutic strategy.^2^^,^^3^

Radiofrequency (RF) catheter ablation has conventionally served as the cornerstone of PVI.^4^^,^^5^ One of the most advanced RF ablation (RFA) technologies is the very high-power short-duration (vHPSD) ablation, applying 90 W power for 4 seconds per ablation point.^6^^,^^7^ The efficacy and safety of vHPSD ablation have been extensively studied.^8^, ^9^, ^10^, ^11^

However, the evolution of ablation technologies has introduced pulsed-field ablation (PFA) with a promising efficacy and safety profile.^12^ Importantly, PFA technologies differ, with the Farapulse system (Boston Scientific, Corp, Marlborough, MA) having the most substantial data. In this article, the term PFA refers exclusively to the Farapulse system. PFA offers similar efficacy and safety as thermal ablation with shorter skin-to-skin procedural times.^13^, ^14^, ^15^ The adoption of PFA necessitates deeper sedation (eg, deep sedation or general anesthesia), diverging from the conscious sedation commonly used in RF procedures.^13^^,^^16^ This shift in sedation practice—requiring deeper anesthesia such as deep sedation or general anesthesia—raises the possibility of prolonging the total time spent in the operating room (OR) (ie, room time), mainly owing to anesthesia-related periods such as induction and emergence.

Importantly, room time represents a comprehensive and clinically relevant measure of procedural efficiency, given that it incorporates not only the technical ablation phase but also patient preparation, anesthesia management, and procedural wrap-up. In high-volume electrophysiology (EP) laboratories, minimizing room time directly affects turnover rates, resource utilization, and staff workload. Despite its practical importance, room time has rarely been the focus of previous comparative studies between ablation technologies.

“Procedure time” usually refers to the skin-to-skin time of the ablation, and the abovementioned procedural phases are often not considered.^16^ However, room time might offer a more objective measurement to compare different technologies’ workflow efficiency.

To the best of our knowledge, no study has compared the fastest RF technology (vHPSD) with PFA in terms of room time. Understanding the detailed temporal aspects of these procedures is paramount for optimizing workflow efficiency, resource utilization, and, ultimately, patient care.

We aimed to conduct a prospective study analyzing the duration of each procedural subsection of both PFA and vHPSD procedures.

## Methods

### Patient population

Consecutive patients who underwent either point-by-point RF catheter ablation using the QDOT Micro catheter (Biosense Webster, Inc, Irvine, CA) in vHPSD mode or PFA with the Farapulse system (Boston Scientific, Corp) for AF at the Heart and Vascular Center of Semmelweis University, Budapest, Hungary, from November 2023 to July 2024 were prospectively included in the present study. The inclusion and exclusion criteria are detailed in the Supplemental Appendix (Supplemental Methods and Supplemental Table 1). All patients agreed to the ablation procedure and provided a written informed consent to data collection and analysis. Ethics approval was granted by the Medical Research Council of the Ministry of Interior (no.: BM/5584-1/2024) and was conducted in accordance with the principles outlined in the Declaration of Helsinki.

### Sedation protocol

We defined the degree of sedation according to previous definitions from different societies of anesthesiology.^17^ Although level 2 is typically referred to as moderate sedation in anesthesiology, we will use the term conscious sedation for the remainder of this manuscript, given that the latter is generally used in EP publications.

RFAs were planned to be performed under conscious sedation using low-dose propofol, fentanyl, and midazolam. If conscious sedation was not satisfactory, drug doses were titrated upward to achieve deep sedation. PFA procedures were mainly performed under deep sedation, except for a few high-risk cases in which general anesthesia was chosen.

Deep sedation was achieved using intravenous 1% propofol (10 mg/mL), administered via continuous infusion at a rate of 25 to 35 mL/h supplemented with 30 to 60 mg propofol boluses and with 25 to 75 μg fentanyl boluses. Deep sedation was carefully titrated until the patient no longer responded to mild pain stimuli while maintaining a patent airway with an oropharyngeal airway (Mayo or Guedel airway). In rare cases, a midazolam bolus of 5 mg was also administered.

### Ablation procedures

**Figure 1:**
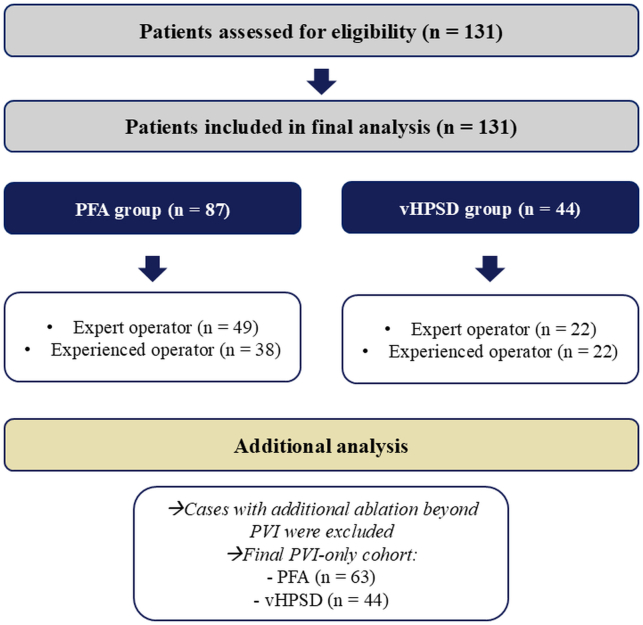
Patient flow diagram. All patients assessed for eligibility (n = 131) were included and assigned to either the PFA or the vHPSD group. Procedures were performed by expert or experienced operators. For the secondary analysis, cases involving ablation beyond PVI were excluded, yielding a final PVI-only cohort. PFA = pulsed-field ablation; PVI = pulmonary vein isolation; vHPSD = very high-power short-duration.

The indications for AF ablation were in line with the current guidelines.^1^^,^^2^ Preprocedural cardiac CT was routinely performed in all patients to exclude left atrial (LA) thrombus. The same 5 physicians performed PFA and RF-based AF ablation procedures, all of whom were experienced in both techniques and had extensively participated in both types of ablations previously. The operators were divided into 2 groups based on their number of lifetime AF ablations: experienced (300–1000 cases) and expert physicians (>1000 cases). The choice of ablation modality was based on shared decision making between the patient and the physician, with the physician’s preference playing an important role. Patient allocation and operator distribution across ablation groups are presented in Figure 1.

#### vHPSD RFA

As stated in the Sedation protocol section, RFA procedures were planned to be performed under conscious sedation using propofol, fentanyl, and midazolam. If this was not sufficient for pain relief or adequate patient compliance, we converted to deep sedation with uptitration of drug doses (for further details, see the Sedation protocol section). After femoral venous access, double transseptal puncture was performed under fluoroscopy guidance and pressure monitoring to access the LA. In some cases, intracardiac echocardiography (ICE) was also used. Intraprocedural anticoagulation was achieved with heparin to maintain a target activated clotting time of 350 seconds. After transseptal puncture, a fast anatomic map of the LA was created using the CARTO mapping system (CARTO3, Biosense Webster, Inc) and a multipolar mapping catheter (Lasso Nav Eco or PentaRay Nav Eco, Biosense Webster, Inc). RFA was performed with the QDOT Micro ablation catheter (Biosense Webster, Inc) using the Agilis NxT steerable sheath (Abbott) and an nGEN generator exclusively in QMODE+ setting (ie, 90 W/4 s). Point-by-point PVI was done with a targeted interlesion distance of 5 mm posteriorly and <5 mm anteriorly, as described previously by our group.^11^ No additional RFA was performed beyond PVI. After the ablation, pulmonary vein (PV) disconnection was evaluated in all patients. First-pass isolation (FPI) was defined as the presence of entrance and exit blocks after completing the first-pass circumferential ablation around the antra of the ipsilateral PVs. In the absence of FPI, additional RFA was done until a bidirectional block was achieved for all PVs. All patients without complications were discharged the day after the procedure.

#### PFA

During PFA procedures, sedation was performed as described in the Sedation protocol section. The Farapulse system (Boston Scientific, Corp) was used for PFA in our study. Owing to local insurance policies, some PFA cases were redo AF ablations. All redo cases involved the reisolation of all PVs as part of the standardized protocol. Similar to the RFA procedures, the procedures were performed using femoral venous access. In contrast, only a single transseptal puncture was performed during PFA procedures. In the same manner as for RFA, intravenous heparin was used to achieve a target activated clotting time of 350 seconds after accessing the LA. The Farawave ablation catheter (Boston Scientific, Corp) was introduced through the Faradrive sheath (Boston Scientific, Corp) into the LA and connected to the Farastar ablation generator (Boston Scientific, Corp). Subsequently, PVI was performed with PFA as follows: 4–4 applications in basket and flower configurations, with a 30-degree catheter rotation after 2 applications in each configuration. Given that there was no contact force feedback with this system at the time of the study, ICE and fluoroscopy were used to evaluate catheter-tissue contact, position, and PV anatomy. As stated, a complete re-PVI was performed with PFA for reinforcement in all redo cases. Therefore, all PFA cases involved a full PVI. PFA was also used in some cases to perform ablation beyond PVI (posterior wall, mitral isthmus, roof isolation, etc) based on the individual judgment of the operating physician. Given that electrical signals immediately disappear after pulsed-field energy delivery, the postablation waiting period and repeated evaluation of PV disconnection have questionable roles in the case of PFA.^18^ Thus, we find it reasonable to evaluate the success of PVI only after the ablation of the given PV, and no repeated check was performed at the end of the procedure. Anesthesia was suspended at the start of ablation of the last PV. Once awake and assessed as neurologically intact, patients were transferred to the inpatient ward. All patients without complications were discharged the day after the procedure.

#### Follow-up and 6-month recurrence definition

After the procedure, outpatient follow-up visits were scheduled at 3 and 6 months, including clinical evaluation and 24-hour Holter electrocardiographic monitoring. Additional visits were arranged if patients reported symptoms suggestive of arrhythmia. Recurrence of AF was defined as any episode of atrial tachyarrhythmia lasting more than 30 seconds, documented by either standard electrocardiographic or 24-hour Holter monitoring. Based on this definition, the 6-month recurrence rate was compared between the 2 groups.

### Definitions of the periods studied

All patients’ total time in the OR (ie, EP laboratory) and the time spent in each subsection of the procedure were measured with certified stopwatches. The time interval definitions are given below, expressed in minutes throughout the manuscript.

Door-in to puncture time: time elapsed between entering the OR (ie, crossing the OR door inward) and the first puncture of the femoral vein.

Puncture to transseptal time: time elapsed between the first femoral vein puncture and the transseptal puncture (ie, the guiding wire is confirmed to be in one of the PVs on fluoroscopy or ICE).

Mapping time: time taken to create the electroanatomic map of the LA. This was only applicable during RFA.

Transseptal to first ablation time: time elapsed between the transseptal puncture and the first ablation lesion created.

Ablation time: time elapsed between the first and the last ablation lesion.

Last ablation to sheath-out time: time elapsed between the last ablation lesion and removing the last catheter sheath.

Sheath-out to door-out time: time elapsed between removing the last catheter sheath and leaving the OR (ie, crossing the OR door outward).

LA dwell time: the time elapsed between the successful transseptal puncture (ie, the guiding wire is confirmed to be in one of the PVs) and the last device leaving the LA cavity.

Procedure time: the total duration of the entire invasive procedure from the first femoral puncture to the removal of the last catheter sheath (ie, skin-to-skin time).

Room time: the total time spent in the OR by a patient, from the moment they entered the OR to when they left. Therefore, besides skin-to-skin procedure time, it also incorporates patient preparation, anesthesia phases, and bringing the patient out of the laboratory.

Fluoroscopy time: the duration of fluoroscopy used during the procedure.

### Statistical analysis

The normality of the parameters was assessed using the Shapiro-Wilk test. Continuous variables are reported as mean ± standard deviation or median with interquartile range (25th–75th percentiles), depending on the distribution. For group comparisons, the Student *t* test was used for normally distributed data, whereas the Mann-Whitney U test was used for non-normally distributed data. Categorical variables are expressed as frequencies and percentages. Contingency analysis was conducted using Pearson’s χ^2^ test. Two-tailed *P* < .05 was considered significant. Statistical analyses were performed using Stata 17 (StataCorp LLC, College Station, TX) and GraphPad Prism 10 software products (GraphPad Software Inc, Boston, MA).

## Results

### Characteristics of the study population

Data from 131 patients were analyzed. Eighty-seven patients (66%) underwent PFA, whereas 44 patients (34%) had vHPSD RFA. The mean age was 66 years (55–71), and 33.6% were female. The only significant difference between the PFA and vHPSD population was the previous PVI (21.8% vs 0%, respectively, *P* = .001) for the reason explained earlier. Baseline characteristics of the study population are presented in Table 1.

**Table 1:** Baseline characteristics of the study population and subpopulations

Variable	All patients (N = 131)	PFA (n = 87)	vHPSD (n = 44)	*P* value
Age (y)	66 (55–71)	65 (55–71)	69 (54.8–73)	.127
Women, n (%)	44 (33.6)	30 (34.5)	14 (31.8)	.093
BMI (kg/m^2^)	27.8 (25.5–31.8)	28.5 (25.5–32)	27.7 (25.2–30.7)	.479
AF type				
Paroxysmal, n (%)	86 (65.6)	52 (59.8)	34 (77.3)	.096
Persistent, n (%)	42 (32.1)	32 (36.8)	10 (22.7)	
Long-standing persistent, n (%)	3 (2.3)	3 (3.4)	0 (0)	
Hypertension, n (%)	94 (71.8)	64 (73.6)	30 (68.2)	.518
Diabetes, n (%)	22 (16.8)	18 (20.7)	4 (9.1)	.088
Hyperlipidemia, n (%)	61 (46.6)	44 (50.6)	17 (38.6)	.176
Thyroid gland disease, n (%)	15 (11.5)	9 (10.3)	6 (13.6)	.545
CAD, n (%)	25 (19.1)	16 (18.4)	9 (20.5)	.776
Previous stroke/TIA, n (%)	8 (6.1)	7 (8.0)	1 (2.3)	.192
PAD, n (%)	3 (2.2)	3 (3.4)	0 (0)	.213
Previous PVI, n (%)	19 (14.5)	19 (21.8)	0 (0)	.001∗
LVEF (%)	57 (52–63)	55 (51–61)	59 (54–65)	.142
iLAV (mL/m^2^)	52.7 (40.5–62.7)	54.7 (42.4–65.4)	49 (38.6–57.9)	.139
LA transverse diameter (mm)	45.1 ± 7.5	46.1 ± 7.4	43.3 ± 7.5	.052
LA longitudinal diameter (mm)	55 (50–60)	56 (51–60)	54 (48–58.3)	.133

AF = atrial fibrillation; BMI = body mass index; CAD = coronary artery disease; iLAV = body surface area–indexed left atrial volume; LA = left atrial; LVEF = left ventricular ejection fraction; PAD = peripheral artery disease; PFA = pulsed-field ablation; PVI = pulmonary vein isolation; TIA = transient ischemic attack; vHPSD = very high-power short-duration.

∗ Statistically significant.

### Periods and their differences and procedural characteristics

Room times with PFA were significantly shorter than vHPSD (71 [64–80] vs 88 [75–99.8] minutes, respectively, *P* < .001). Furthermore, almost all procedural phases were significantly shorter in PFA procedures than RFA (all *P* < .001), except the door-in to puncture and the puncture to transseptal time (all *P* > .05). Meanwhile, the sheath-out to door-out time was significantly longer in PFA cases (*P* < .001).

**Figure 2:**
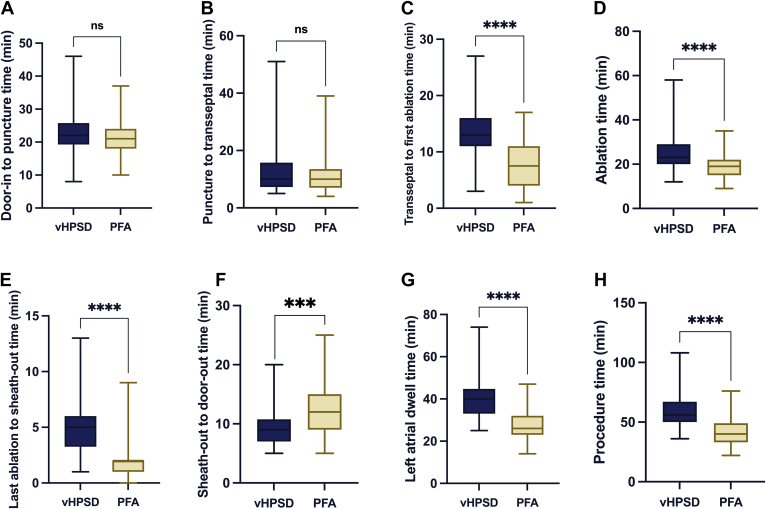
Time measurements and differences of procedural subsections. Comparing time across procedural subsections: vHPSD (*blue*) vs PFA (*yellow*). **A:** Door-in to puncture time, with no difference observed. **B:** Puncture to transseptal time; again, no difference. Significant reductions are seen in transseptal to first ablation time (**C**) and ablation time (**D**) with PFA. **E:** A significant decrease in the last ablation to sheath-out time with PFA. **F:** The sheath-out to door-out time was significantly lower in the case of vHPSD ablation. **G and H:** Significant reductions in left atrial dwell time and procedure time with PFA. These findings indicate that PFA offers improved efficiency over vHPSD in almost all key procedural stages. ns = *P* > .05; ∗∗∗*P* < .001; ∗∗∗∗*P* < .0001. min = minutes; ns = not significant; PFA = pulsed-field ablation; vHPSD = very high-power short-duration.

The times of procedural subsections are presented in Table 2 and Figure 2.

**Table 2:** Procedural characteristics

Variable	All patients (N = 131)	PFA (n = 87)	vHPSD (n = 44)	*P* value
Door-in to puncture time (min)	21 (18–24.5)	21 (18–24)	22 (19.3–25.8)	.222
Puncture to transseptal time (min)	10 (7–14)	10 (7–13.5)	10 (7.3–15.8)	.657
Mapping time (min)	-	-	9.3 ± 3.5	-
Transseptal to first ablation time (min)	9.9 ± 5.2	7.7 ± 4.3	13.9 ± 4.4	<.001∗
Ablation time (min)	20 (16–25)	19 (15–22)	23 (20–29)	<.001∗
Last ablation to sheath-out time (min)	2 (1–4.5)	2 (1–2)	5 (3.3–6)	<.001∗
Sheath-out to door-out time (min)	10 (8–14)	12 (9–15)	9 (7–10.8)	<.001∗
Left atrial dwell time (min)	29 (24–37.5)	26 (23–32)	40 (33–44.8)	<.001∗
Procedure time (min)	43.5 (37–56)	40 (33–49)	56 (50–67)	<.001∗
Room time (min)	75 (67.8–88)	71 (64–80)	88 (75–99.8)	<.001∗
Anesthesia types				
Conscious sedation, n (%)	39 (29.8)	0 (0)	39 (88.6)	<.001∗
Deep sedation, n (%)	88 (67.2)	83 (95.4)	5 (11.4)	
General anesthesia, n (%)	4 (3.1)	4 (4.6)	0 (0)	
Complications† and hospital stay				
Total, n (%)	4 (3.1)	3 (3.4)	1 (2.3)	.697
Minor, n (%)	3 (2.3)	2 (2.3)	1 (2.3)	.993
Major, n (%)	1 (0.8)	1 (1.1)	0 (0)	.320
Hospital stay (d)	1 (1–1)	1 (1–1)	1 (1–1)	.320
Recurrence rate				
6-mo recurrence rate, n (%)	21 (16.0)	13 (14.9)	8 (18.2)	.646

min = minutes; PFA = pulsed-field ablation; vHPSD = very-high-power short-duration.

∗ Statistically significant.

† See Section 3.2 for a detailed description.

**Figure 3:**
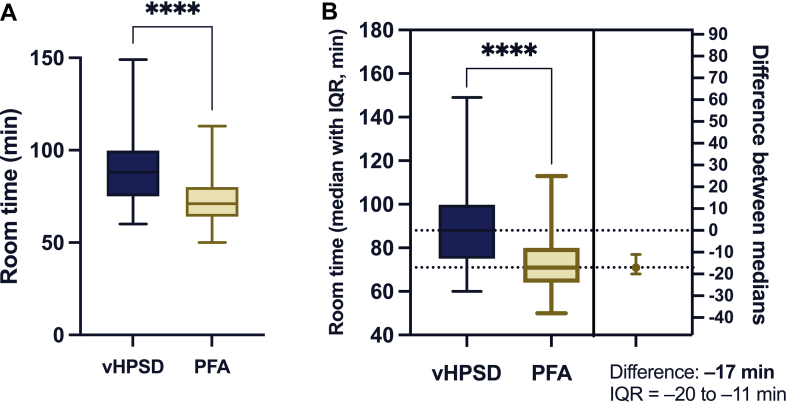
PFA vs vHPSD room times and their differences. Comparing the room times (defined as the total time spent in the OR by a patient) of vHPSD and PFA procedures. **A:** A significant reduction in room time with PFA (*yellow*) compared with vHPSD (*blue*). **B:** A 17-minute median reduction favoring PFA (IQR −20 to −11 minutes). ∗∗∗∗*P* < .0001. IQR = interquartile range; min = minutes; OR = operating room; PFA = pulsed-field ablation; vHPSD = very high-power short-duration.

The difference in the room time of PFA and vHPSD is presented in Figure 3.

The potential impact of electroanatomic mapping on PFA room time was assessed in Supplemental Results subsection 2.1.

The implemented anesthetic modalities are also presented in Table 2.

Fluoroscopy time (8 [5.8–12] vs 4.9 [2.5–6.6] minutes, *P* < .001), absorbed dose (15 [9–23] vs 8 [3–14.5] mGy, *P* < .001), and dose area product (180.1 [104.5–282.3] vs 91.1 [45.1–170.9] μGym^2^, *P* < .001) were significantly higher in the PFA group than the vHPSD group.

Twenty-four patients (27.6%) underwent additional ablation beyond PVI in the PFA group, whereas none had additional ablation with vHPSD. A detailed description of additional ablation is presented in Supplemental Table 2.

Data regarding transseptal puncture guidance are presented in Supplemental Table 3.

FPI with vHPSD was achieved in 151 PVs (89.3%); bilateral FPI was achieved in 35 patients (79.5%).

Data on starting rhythms at the beginning of catheter ablation and the mechanism of sinus rhythm restoration are presented in the Supplemental Appendix (Supplemental Results subsection 2.2 and Supplemental Table 4).

The 2 groups had no significant difference in the PV anatomy variation distribution (*P* > .05). For detailed information, see Table 3.

**Table 3:** Pulmonary vein anatomy variations

Anatomical variation	All patients (N = 131)	PFA (n = 87)	vHPSD (n = 44)	*P* value
Normal PV anatomy, n (%)	108 (82.4)	71 (81.6)	37 (84.1)	.803
LSCT, n (%)	5 (3.8)	3 (3.5)	2 (4.5)	
LLCT, n (%)	15 (11.5)	10 (11.5)	5 (11.4)	
RMPV, n (%)	2 (1.5)	2 (2.3)	0 (0)	
LSCT + RCT, n (%)	1 (0.8)	1 (1.1)	0 (0)	

LLCT = left long common trunk; LSCT = left short common trunk; PFA = pulsed-field ablation; PV = pulmonary vein; RCT = right common trunk; RMPV = right middle PV; vHPSD = very high-power short-duration.

Four complications occurred during our study without a significant difference between the groups (*P* > .05). There was 1 groin hematoma in both the PFA and vHPSD groups that did not require further intervention. In 1 PFA case, extensive ST elevation occurred. Coronary angiography was performed, which did not confirm coronary artery disease, and the ST elevation resolved spontaneously within a few minutes without residual symptoms. The patient resolved without sequelae and was discharged the next day. One major complication occurred in a PFA patient who developed an arteriovenous fistula at the site of femoral puncture, requiring vascular surgery the day after the ablation. There was no significant difference in hospital stay between groups (median in both groups 1 day [interquartile range 1–1], *P* = .320). At the 6-month follow-up, AF recurrence occurred in 16.0% of patients, with no significant difference between groups (*P* = .646) (Table 2).

Additional analysis comparing PVI-only PFA with PVI-only vHPSD cases showed similarly significant results (for details, see Supplemental Results subsection 2.3, Supplemental Tables 5–9, and Supplemental Figure 1).

### Subgroup analysis of experienced and expert electrophysiologists

A subgroup analysis was conducted based on the operators’ level of experience. Room times of procedures performed by expert operators were significantly shorter than experienced operators, both with PFA (69 [60.3–74] vs 76 [66–87.3] minutes, respectively, *P* = .003) and vHPSD (80 [72–87] vs 97.5 [88.8–114.5] minutes, respectively, *P* < .001). The difference between room times of expert and experienced operators was significantly lower in the case of PFA (median difference, 7 [6–13] for PFA vs 18 [17–28] minutes for vHPSD).

**Figure 4:**
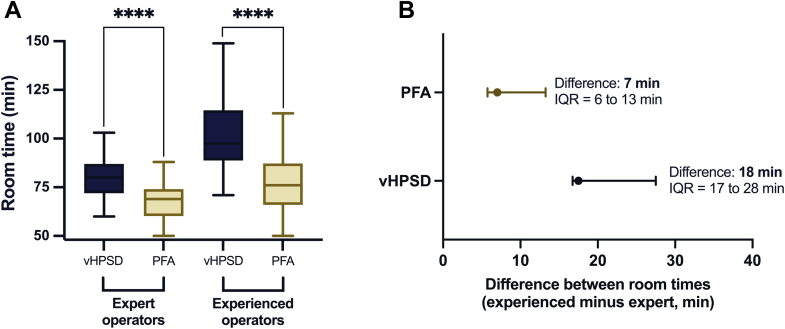
Subgroup analysis of room times based on operator experience. Comparing room times (defined as the total time spent in the OR by a patient) between PFA (*yellow*) and vHPSD (*blue*) by operator experience. **A:** Both expert operators (those with a lifetime experience of >1000 AF cases) and experienced operators (those with 300–1000 AF cases) achieved significantly shorter room time with PFA than vHPSD. **B:** The difference in room times between experienced and expert operators, showing a 7-minute difference for PFA (IQR 6–13 minutes) and an 18-minute difference for vHPSD ablation (IQR 17–28 minutes) in favor of experts. ∗∗∗∗*P* < .0001. AF = atrial fibrillation; IQR = interquartile range; min = minutes; OR = operating room; PFA = pulsed-field ablation; vHPSD = very high-power short-duration.

When comparing within subgroups, both had significantly shorter PFA room times than vHPSD, especially in the experienced subgroup (cases done by expert operators, 69 [60.3–74] vs 80 [72–87] minutes, respectively, *P* < .001; cases performed by experienced operators, 76 [66–87.3] vs 97.5 [88.8–114.5] minutes, respectively, *P* < .001) (Figure 4).

## Discussion

### Main findings

Our study shows that the total time spent by the patient in the OR (ie, room time) of PFA is significantly shorter than the room time of the fastest RF technology (ie, vHPSD ablation) in the case of AF ablation, without difference in 6-month recurrence rates. Moreover, we have found that the room time of PFA procedures is less influenced by the operator’s level of experience.

### Comparison of room and other times with the 2 modalities

When comparing the fastest currently available RF technology (vHPSD, 90 W/4 s RFA) with PFA,^8^ we found a median 17-minute reduction in the total time spent in the OR (room time) in favor of PFA. In particular, a complete PFA procedure could be performed in only 71 minutes from OR entry to exit compared with 88 minutes in the case of vHPSD. This efficiency provides an opportunity to further optimize time management in the EP laboratory. We are the first to present real-world data demonstrating the time savings achievable in room time with PFA despite the need for deeper sedation compared with RFA. This is of increased importance because a definite rise in AF incidence is predicted in the coming decades; therefore, it is essential to identify strategies to deliver standardized yet time-efficient care to as many patients as possible.^1^

The procedure time was also significantly longer for the vHPSD setting than PFA (56 vs 40 minutes). When considering the differences, one must be aware that even the RFA procedures were relatively fast, with an average of 56 minutes of skin-to-skin time.^9^^,^^19^, ^20^, ^21^ There are 2 main factors causing the difference between vHPSD and PFA. First, there is no need for electroanatomic mapping with PFA, which is also indicated by the significant difference in the transseptal to first ablation time (8 [PFA] vs 14 minutes [vHPSD]). Second, catheter navigation and maneuvering are more straightforward with the single-shot, well-designed Farapulse device than point-by-point ablation, as illustrated by the significant difference in ablation times (19 [PFA] vs 23 minutes [vHPSD]). The difference favoring the quickness of PFA is also apparent when comparing the expert and experienced operators; although the room time difference was clinically negligible (7 minutes) in the case of PFA, it was clinically meaningful (18 minutes) in the case of vHPSD.

The reductions shown by our results in skin-to-skin procedure time and LA dwell time align with previous studies comparing thermal ablation with PFA. In the ADVENT trial, the Farapulse system demonstrated a 29% reduction in LA dwell times and a 14% reduction in procedure times vs thermal ablation modalities. The mean total procedure time was 105.8 ± 29.4 minutes for patients who underwent PFA and 123.1 ± 42.1 minutes for patients who underwent thermal ablation.^22^ One of the most recent single-center comparisons between cryoablation and PFA confirmed a 30% reduction in procedure times with PFA. The median procedure time was 34.5 minutes with PFA compared with 50 minutes with cryoenergy.^23^ Our results show that an even greater reduction of approximately 29% (compared with the ADVENT trial’s 14%) can be achieved with PFA than the fastest RFA technology regarding procedure time. At the same time, the reduction of LA dwell time was also higher (35%).

Notably, despite the additional steps of positioning the CARTO patches and preparing 2 transseptal sheaths in vHPSD procedures, the door-in to puncture time did not significantly differ between PFA and vHPSD procedures (21 [PFA] vs 22 minutes [vHPSD]). This can be attributed to our highly experienced team of scrub nurses and technicians involved in the procedures.

Regarding the use of ionizing radiation, PFA procedures resulted in longer fluoroscopy time and consequently higher absorbed dose and dose area product than vHPSD ablation. In the ADVENT trial, PFA was associated with similarly higher radiation doses than thermal ablation.^22^ This is directly linked to the absence of LA mapping and the consequent need for fluoroscopy guidance throughout PFA cases. However, although PFA involves significantly higher radiation exposure, it is important to note that these remain well below those of other catheter-based cardiology procedures, such as invasive coronary angiography.^24^^,^^25^

An important difference between the 2 modalities is that, because of the pain caused by applications, PFA warrants a higher level of anesthesia throughout the procedure,^14^^,^^26^ for example, deep sedation or general anesthesia.^16^^,^^27^, ^28^, ^29^ This is in contrast with RFA, where, in most cases, AF ablation can be performed under conscious sedation. The difference in anesthesia significantly affects procedure times.^30^ However, previous studies have compared different ablation modalities and types of anesthesia based solely on procedure time. We hypothesized that although procedure times may be shorter with deeper anesthesia, the induction and emergence periods may prolong the room time.^16^^,^^28^ Consequently, a more extended recovery period was expected at the end of the PFA cases. Indeed, our results reflect this difference: sheath-out to door-out time was significantly longer for PFA cases than vHPSD cases (12 [PFA] vs 9 minutes [vHPSD]). Nevertheless, this was compensated by meaningfully shorter skin-to-skin procedure times, leading to a significantly shorter room time with PFA (71 [PFA] vs 88 minutes [vHPSD]).

Although not directly measured, based on our results, by saving nearly 17 minutes per intervention, PFA allows for an extra procedure after every 4 cases compared with vHPSD ablation, potentially improving overall laboratory efficiency and patient throughput without compromising 6-month recurrence rates. Furthermore, this was achieved with more than a quarter of PFA cases (27.6%) involving additional ablation beyond PVI, whereas vHPSD cases were limited to PVI. Hence, we believe that an even more pronounced difference could be achieved in a PVI-only comparison. When comparing expert and experienced operators, the difference in room time was smaller for PFA (69 vs 76 minutes; 7-minute difference, clinically negligible) than for vHPSD (80 vs 98 minutes; 18-minute difference, clinically more meaningful). This suggests that PFA procedures are technically less challenging or operator dependent.

Although we did not perform electroanatomic mapping during PFA procedures at our center, we acknowledge that the use of mapping in PFA is increasingly adopted in clinical practice. According to the ADVENT trial,^22^ the mean mapping time in PFA procedures was 3.8 ± 6.0 minutes. Even if this value were added to the PFA group’s room time in our study, the adjusted mean would remain below that of the vHPSD group, supporting the notion that PFA maintains a room time advantage even when electroanatomic mapping is incorporated.

### Comparison of safety and complication profiles

Our study found no difference in complication rates, with 3 minor and 1 major complication reported, and there was no difference in the length of hospital stay between the 2 groups. These numbers align with previous data: the complication rates with PFA seem similar to those of conventional thermal ablation technologies such as RF and cryoablation.^31^ The MANIFEST-PF survey reported a 1.6% rate for major and 3.9% for minor complications, comparable with conventional thermal energy sources.^14^ In the Q-FFICIENCY study, after vHPSD ablation, the primary adverse event rate was reported to be 3.6%.^32^

### Subgroup analysis based on operator experience

We also examined the impact of operator expertise on the comparative time efficiency of the 2 approaches. Subgroup division was based on procedural volume determined by the arbitrary number of 1000 successful cases.

Room time with PFA performed by expert operators was shorter than the PFA done by experienced operators (69 vs 76 minutes). We found similar results among the vHPSD cases (80 vs 98 minutes). However, although with vHPSD the procedures of experts were substantially faster (18 minutes), the difference was not clinically relevant in the case of PFA (7 minutes).

These insights demonstrate the potential role of PFA in standardizing procedural efficiency across operators of varying experience levels. Similar conclusions were shown in the EU-PORIA registry, where procedural and outcome data were examined based on operators’ experience with AF ablation, showing no significant differences in procedural metrics or complications.^13^ Standardization is of particular importance in the case of AF ablation because it is the most commonly performed EP procedure worldwide.^1^^,^^2^

### Limitations

The current results are based on a single-center, observational, and nonrandomized study, which carries inherent limitations. Although randomization was not performed, the validity of our results comparing the 2 modalities is strengthened by the absence of significant differences between the characteristics of the 2 populations. The only significant difference between PFA and vHPSD patients was the prevalence of previous PVI (22% vs 0%) related to the local insurance policies.

Procedures were performed by multiple operators with varying levels of experience, and the sample size is relatively small and differs between the subgroups. Procedure time may have been influenced by the transseptal access strategy, which was determined by the ablation modality: single puncture for PFA and double puncture for vHPSD ablation.

We did not report the long-term efficacy of the procedures. However, this was investigated by previous trials for both PFA and vHPSD.

Although the current findings are promising, PFA’s long-term reliability and effectiveness are still awaited, which may introduce some caution to our conclusions.

Moreover, this study specifically focuses on the Farapulse PFA. In contrast, several vendors are introducing novel PFA systems to the market, some of which include integration with electroanatomic mapping systems.

## Conclusion

Our results showed that, in the case of AF ablation, the total time spent in the OR (room time) during PFA procedures is significantly shorter than the latest and fastest RF technology (vHPSD) despite the need for deeper sedation. Importantly, there was no significant difference in 6-month AF recurrence rates between the 2 groups. Our findings also indicated that PFA may be less operator dependent than RFA. Our data suggest that PFA may further improve the time efficiency of AF ablation, even compared with the fastest RF technology.
