# Supplementary material for: Comparison of room times between pulsed-field ablation and very high-power short-duration ablation
Source: Heart Rhythm O2. 2025 Jul 18;6(10):1546–55. doi: 10.1016/j.hroo.2025.07.008 (PMC12570189; doi:10.1016/j.hroo.2025.07.008)
Supplement: Supplementary Data [file mmc1.docx]

**Supplemental Appendix**

**Full Title**:

Comparison of Room Times between Pulsed-Field Ablation and Very High-Power Short-Duration Ablation

**Author List**:

Gábor Orbán^a^*****, MD; Márton Boga^a^, MD; Zoltán Salló^a^, MD; István Osztheimer^a^, MD, PhD; Klaudia Vivien Nagy^a^, MD, PhD; Péter Perge^a^, MD, PhD; Edit Tanai^a^, MD; Bence Czumbel^a^; Bertalan Bakán^a^; Ferenc Komlósi^a^, MD; Patrik Tóth^a^, MD; Arnold Béla Ferencz^a^, MD; Béla Merkely^a^, MD, PhD, DSc; László Gellér^a^, MD, PhD, DSc; Nándor Szegedi^a^, MD, PhD

**Authors' Affiliations**:

^a^Heart and Vascular Center, Semmelweis University, 68 Városmajor Street, H-1122 Budapest, Hungary

***Corresponding author**:

- Full name: Gábor Orbán, MD
- Postal address: Városmajor u. 68, 1122 Budapest, Hungary
- E-mail address: orbn.gbor@gmail.com

**Supplemental Appendix**:

Supplemental Methods: Page 3

Supplemental Results: Pages 4-6

Supplemental Tables 1-9: Pages 7-18

- Supplemental Table 1: Detailed patient inclusion and exclusion criteria (Page 7)
- Supplemental Table 2: Additional ablations beyond pulmonary vein isolation (Pages 8-9)
- Supplemental Table 3: Imaging modalities for transseptal puncture guidance (Page 10)
- Supplemental Table 4: Starting rhythm and mechanism of sinus rhythm restoration (Page 11)
- Supplemental Table 5: Baseline characteristics of the PVI-only subpopulation (Pages 12-13)
- Supplemental Table 6: Pulmonary vein anatomy variations in the PVI-only subpopulation (Page 14)
- Supplemental Table 7: Starting rhythm and mechanism of sinus rhythm restoration in the PVI-only subpopulation (Page 15)
- Supplemental Table 8: Imaging modalities for transseptal puncture guidance in the PVI-only subpopulation (Page 16)
- Supplemental Table 9: Procedural characteristics of the PVI-only subpopulation (Pages 17-18)

Supplemental Figure 1: Analysis of room times based on operator experience in the PVI-only PFA population (Pages 19-20)

**1. Supplemental Methods:**

Supplemental Table 1 outlines the study’s inclusion and exclusion criteria for patient selection in the study.

**2. Supplemental Results:**

**2.1. Sensitivity analysis: Impact of potential electroanatomical mapping on PFA room time**

In response to the reviewer’s suggestion, we conducted a sensitivity analysis to evaluate the potential impact of 3D electroanatomical mapping on the room time of PFA cases. Although 3D mapping was not utilized during PFA procedures in our study, we acknowledge its increasing integration into PFA workflows in many centers, owing to its procedural and safety-related advantages.

For this analysis, we added the median mapping time observed in the RF group (9 minutes) to the original room times of the PFA group. Following this adjustment, the room time in the adjusted PFA group was 80 (73-89) minutes. Despite this modification, the difference between the adjusted PFA room time and the RF group’s room time [88 (75-99,8) minutes] remained statistically significant (p=0.0151).

**2.2. Starting rhythm and mechanisms of sinus rhythm restoration**

Most of the patients were in sinus rhythm (SR) at the start of the procedure, and there was no difference in this aspect between the two groups (p=0.271). All patients left the operating room with SR. There was no significant difference in the mechanism of SR restoration between the pulsed-field ablation (PFA) and very high-power short-duration (vHPSD) groups (all p>0.05). More information can be found in **Supplemental Table 4**.

**2.3. PVI-only analysis**

We conducted additional analysis excluding cases where additional ablation was performed beyond pulmonary vein isolation (PVI). This meant the exclusion of 24 PFA cases. Similarly to the main population, the only difference found between PVI-only PFA and PVI-only vHPSD patients was the prior PVI (20.6% versus 0%, respectively, p = 0.001) (**Supplemental Table 5**), which is related to the local insurance policies.

Again, in line with the main population, **Supplemental Tables 6-7** show no significant differences in pulmonary vein anatomy, starting rhythms, or mechanism of sinus rhythm restoration between PVI-only PFA and PVI-only vHPSD groups.

Data regarding transseptal puncture guidance in the PVI-only cohort is available in **Supplemental Table 8**.

Analysis of the time periods showed differences similar to those in the main population (**Supplemental Table 9**). Interestingly, the median room time for the PVI-only PFA procedures was one minute longer compared to the room time of the main PFA population [72 (63-83 vs. 71 (64-80) mins, respectively].

Further investigation revealed that 91.7% of additional ablations (22 out of 24 cases) were performed by expert operators (definition of expert and experienced operators can be found in the main text’s **Ablation procedures subsection**). Therefore, by excluding cases with additional ablation, mainly expert cases were excluded. The argument for the increased room time of PVI-only PFA procedures can be that the experts were more efficient with PFA, so that even the expert cases involving additive ablation had shorter room times than the experienced operators' PFA PVI-only cases.

To delve even further, we compared the room times within the PFA PVI-only population between expert and experienced operators. This analysis showed that the median time difference increased from the original seven minutes [overall PFA population: expert room time of 69 (60.3-74) vs. experienced room time of 76 (66-87.3) mins, p=0.003; median difference 7 (6–13) mins] to nine minutes in the PVI-only population [expert PVI-only room time: 67 (60-76) vs. experienced PVI-only room time: 76 (66-87,75) mins, p=0.013; median difference PFA PVI-only: 9 (6-12) mins] (**Supplemental Figure 1**).

**Supplemental Table 1**: Detailed patient inclusion and exclusion criteria

| Inclusion criteria | Symptomatic paroxysmal / persistent / long-standing persistent atrial fibrillation |
| --- | --- |
|  | Voluntary consent to participate |
|  | Age over 18 years |
| Exclusion criteria | Contraindication to ablation |
|  | Contraindication of long-term anticoagulation |
|  | History of cardiac surgery |
|  | Pregnancy |
|  | Active malignancy |
|  | Life expectancy <1 year |
|  | Valvular atrial fibrillation |
|  | Reversible cause of atrial fibrillation (e.g., hyperthyroidism) |

**Supplemental Table 2**: Additional ablations beyond pulmonary vein isolation

|  | **All patients (n = 131)** | **PFA (n = 87)** | **vHPSD (n = 44)** |
| --- | --- | --- | --- |
| No additional ablation (i.e., PVI-only), n (%) | 107 (81.7) | 63 (72.4) | 44 (100) |
| Posterior wall ablation, n (%) | 12 (9.2) | 12 (13.8) | 0 |
| Posterior wall + mitral isthmus ablation, n (%) | 6 (4.6) | 6 (6.9) | 0 |
| Posterior wall + roof ablation, n (%) | 3 (2.3) | 3 (3.4) | 0 |
| Mitral isthmus + roof ablation, n (%) | 1 (0.76) | 1 (1.15) | 0 |
| Posterior wall + mitral isthmus + roof ablation, n (%) | 1 (0.76) | 1 (1.15) | 0 |
| Posterior wall + mitral isthmus + roof + anterior wall ablation, n (%) | 1 (0.76) | 1 (1.15) | 0 |

Abbreviations: PFA, pulsed-field ablation. vHPSD, very high-power short-duration.

**Supplemental Table 3**: Imaging modalities for transseptal puncture guidance

|  | **All patients (n = 131)** | **PFA (n = 87)** | **vHPSD (n = 44)** |
| --- | --- | --- | --- |
| Only fluoroscopy, n (%) | 22 (16.8) | 0 | 22 (50.0) |
| Fluoroscopy + ICE, n (%) | 104 (79.4) | 82 (94.3) | 22 (50.0) |
| Only ICE, n (%) | 5 (3.8) | 5 (5.7) | 0 |

Abbreviations: ICE, intracardiac echocardiography; PFA, pulsed-field ablation; vHPSD, very high-power short-duration.

**Supplemental Table 4**: Starting rhythm and mechanism of sinus rhythm restoration

|  | **All patients (n = 131)** | **PFA (n = 87)** | **vHPSD (n = 44)** | **p-value** |
| --- | --- | --- | --- | --- |
| SR at the beginning of the procedure, n (%) | 97 (74.0) | 61 (70.1) | 36 (81.8) | 0.271 |
| SR restored during ablation, n (%) | 18 (13.7) | 13 (14.9) | 5 (11.4) | 0.53 |
| Successful ECV to SR, n (%) | 24 (18.3) | 13 (14.9) | 11 (25.0) | 0.21 |
| SR at the end of the procedure, n (%) | 131 (100) | 87 (100) | 44 (100) | 1 |

Abbreviations: ECV, electrical cardioversion; PFA, pulsed-field ablation; SR, sinus rhythm; vHPSD, very high-power short-duration.

**Supplemental Table 5**: Baseline characteristics of the PVI-only subpopulation

|  | **PVI-only patients (n = 107)** | **PVI-only PFA (n = 63)** | **PVI-only vHPSD (n = 44)** | **p-value** |
| --- | --- | --- | --- | --- |
| Age (years) | 66 (55-71) | 65 (55-70) | 69 (54.8-73) | 0.106 |
| Female, n (%) | 31 (29) | 17 (27) | 14 (31.8) | 0.588 |
| BMI (kg/m^2^) | 27.8 (25.6-31.8) | 28.4 (25.6-32) | 27.7 (25.2-30.7) | 0.396 |
| **AF type** | | | | |
| Paroxysmal, n (%) | 71 (66.4) | 37 (58.7) | 34 (77.3) | 0.081 |
| Persistent, n (%) | 33 (30.8) | 23 (36.5) | 10 (22.7) |  |
| Long-standing persistent, n (%) | 3 (2.8) | 3 (4.8) | 0 |  |
| Hypertension, n (%) | 76 (71) | 46 (73) | 30 (68.2) | 0.588 |
| Diabetes, n (%) | 17 (15.9) | 13 (20.6) | 4 (9.1) | 0.101 |
| Hyperlipidemia, n (%) | 47 (43.9) | 30 (47.6) | 17 (38.6) | 0.319 |
| Thyroid gland disease,  n (%) | 10 (9.3) | 4 (6.3) | 6 (13.6) | 0.188 |
| CAD, n (%) | 22 (20.6) | 13 (20.6) | 9 (20.5) | 0.982 |
| Prior stroke/TIA, n (%) | 7 (6.5) | 6 (9.5) | 1 (2.3) | 0.136 |
| PAD, n (%) | 3 (2.8) | 3 (4.8) | 0 | 0.142 |
| Prior PVI, n (%) | 13 (12.1) | 13 (20.6) | 0 (0) | **0.001** |
| LVEF (%) | 55 (50-62.8) | 55 (49-60.5) | 59 (54-65) | 0.607 |
| iLAV (ml/m^2^) | 53 (41.3-61.9) | 54.8 (43.7-66.5) | 49 (38.6-57.9) | 0.107 |
| LA transverse diameter (mm) | 44.8 ± 7.3 | 45.9 ± 6.9 | 43.3 ± 7.5 | 0.071 |
| LA longitudinal diameter (mm) | 55 (50-60) | 57 (51-60) | 54 (48-58.3) | 0.072 |

Abbreviations: AF, atrial fibrillation; BMI, body mass index; CAD, coronary artery disease; iLAV, body surface area-indexed left atrial volume; LA, left atrial; LVEF, left ventricular ejection fraction; PAD, peripheral artery disease; PFA, pulsed-field ablation; PVI, pulmonary vein isolation; TIA, transient ischemic attack; vHPSD, very high-power short-duration.

**Supplemental Table 6**: Pulmonary vein anatomy variations in the PVI-only subpopulation

|  | **PVI-only patients (n = 107)** | **PVI-only PFA (n = 63)** | **PVI-only vHPSD (n = 44)** | **p-value** |
| --- | --- | --- | --- | --- |
| Normal PV anatomy, n (%) | 90 (84.1) | 53 (84.1) | 37 (84.1) | 0.636 |
| LSCT, n (%) | 5 (4.7) | 3 (4.8) | 2 (4.5) |  |
| LLCT, n (%) | 9 (8.4) | 4 (6.3) | 5 (11.4) |  |
| RMPV, n (%) | 2 (1.9) | 2 (3.2) | 0 |  |
| LSCT + RCT, n (%) | 1 (0.9) | 1 (1.6) | 0 |  |

Abbreviations: LLCT, left long common trunk; LSCT, left short common trunk; PFA, pulsed-field ablation; PV, pulmonary vein; RCT, right common trunk; RMPV, right middle PV; vHPSD, very high-power short-duration.

**Supplemental Table 7**: Starting rhythm and mechanism of sinus rhythm restoration in the PVI-only subpopulation

|  | **PVI-only patients (n = 107)** | **PVI-only PFA (n = 63)** | **PVI-only vHPSD (n = 44)** | **p-value** |
| --- | --- | --- | --- | --- |
| SR at the beginning of the procedure, n (%) | 83 (77.6) | 47 (74.6) | 36 (81.8) | 0.458 |
| SR restored during ablation, n (%) | 12 (11.2) | 7 (11.1) | 5 (11.4) | 0.991 |
| Successful ECV to SR, n (%) | 21 (19.6) | 10 (15.9) | 11 (25.0) | 0.259 |
| SR at the end of the procedure, n (% | 107 (100) | 63 (100) | 44 (100) | 1 |

Abbreviations: ECV, electrical cardioversion; PFA, pulsed-field ablation; SR, sinus rhythm; vHPSD, very high-power short-duration.

**Supplemental Table 8**: Imaging modalities for transseptal puncture guidance in the PVI-only subpopulation

|  | **PVI-only patients (n = 107)** | **PVI-only PFA (n = 63)** | **PVI-only vHPSD (n = 44)** |
| --- | --- | --- | --- |
| Only fluoroscopy, n (%) | 22 (20.6) | 0 | 22 (50.0) |
| Fluoroscopy + ICE, n (%) | 81 (75.7) | 59 (93.7) | 22 (50.0) |
| Only ICE, n (%) | 4 (3.7) | 4 (6.3) | 0 |

Abbreviations: ICE, intracardiac echocardiography; PFA, pulsed-field ablation; vHPSD, very high-power short-duration.

**Supplemental Table 9**: Procedural characteristics of the PVI-only subpopulation

|  | **PVI-only patients (n = 107)** | **PVI-only PFA (n = 63)** | **PVI-only vHPSD (n = 44)** | **p-value** |
| --- | --- | --- | --- | --- |
| Door-in to puncture time (min) | 21 (18-24) | 20 (16-24) | 22 (19.3-25.8) | 0.088 |
| Puncture to transseptal time (min) | 10 (7-14.3) | 10 (7-14) | 10 (7.3-15.8) | 0.914 |
| Mapping time (min) | - | - | 9.3 ± 3.5 | - |
| Transseptal to first ablation time (min) | 10.3 ± 5.2 | 7.7 ± 4.5 | 13.9 ± 4.4 | **<0.001** |
| Ablation time (min) | 20 (16-25) | 18 (14-23) | 23 (20-29) | **<0.001** |
| Last ablation to sheath out time (min) | 2 (1-5) | 1 (1-2) | 5 (3.3-6) | **<0.001** |
| Sheath out to door out time (min) | 10 (8-14) | 12 (9-15) | 9 (7-10.8) | **<0.001** |
| Left atrial dwell time (min) | 32 (24-40) | 25 (22-32) | 40 (33-44.8) | **<0.001** |
| Procedure time (min) | 45.5 (37.3-57) | 40 (33-49) | 56 (50-67) | **<0.001** |
| Room time (min) | 76 (69-88) | 72 (63-83) | 88 (75-99.8) | **<0.001** |
| **Anaesthesia types** | | | | |
| Conscious sedation, n (%) | 39 (36.4) | 0 | 39 (88.6) | **<0.001** |
| Deep sedation, n (%) | 67 (62.6) | 62 (98.4) | 5 (11.4) |  |
| General anaesthesia, n (%) | 1 (0.93) | 1 (1.6) | 0 |  |

Abbreviations: PFA, pulsed-field ablation; PVI, pulmonary vein isolation; vHPSD, very high-power short-duration.

**Supplemental Figure 1**: Analysis of room times based on operator experience in the PVI-only PFA population


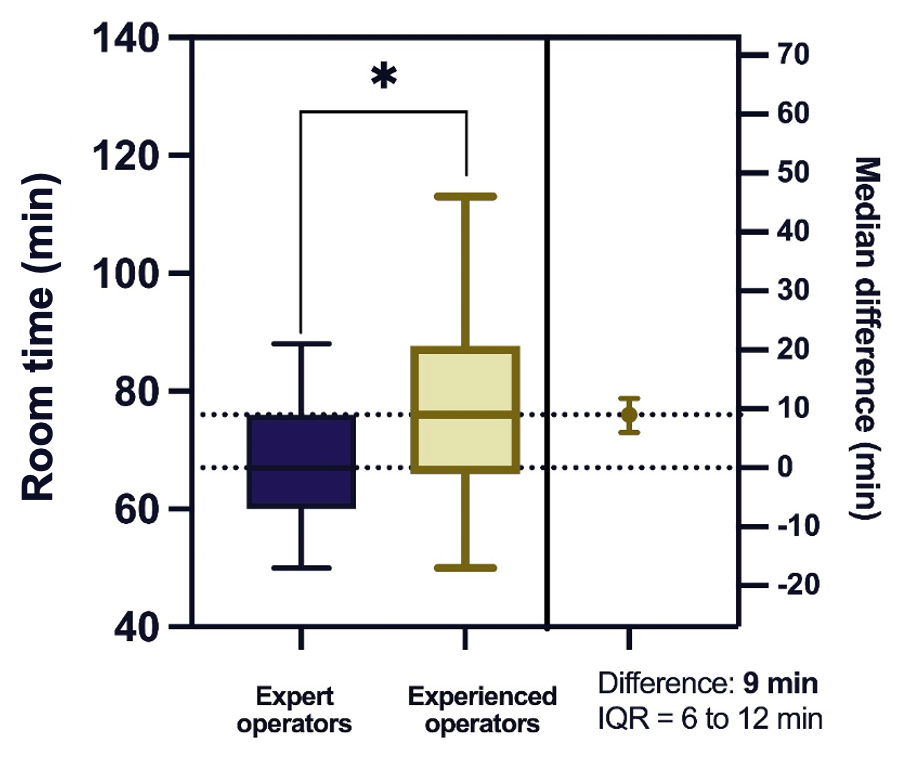


Caption: This figure compares room times (defined as the total time spent in the OR by a patient) in the PVI-only PFA population between two groups of operators: experts and experienced ones. Expert operators, represented in blue, are those with a lifetime experience of over 1,000 AF cases, while experienced operators, shown in yellow, have performed between 300 and 1,000 AF cases. The figure illustrates that expert operators achieved significantly shorter room time, with a median difference of 9 minutes (IQR: 6 to 12 minutes).

Abbreviations: AF, atrial fibrillation; IQR, interquartile range; OR, operating room; PFA, pulsed-field ablation; PVI, pulmonary vein isolation; *=p<0.05.
